# Identification of a Eukaryotic Reductive Dechlorinase and Characterization of Its Mechanism of Action on Its Natural Substrate

**DOI:** 10.1016/j.chembiol.2011.08.003

**Published:** 2011-10-28

**Authors:** Francisco Velazquez, Sew Yu Peak-Chew, Israel S. Fernández, Christopher S. Neumann, Robert R. Kay

**Affiliations:** 1 Laboratory of Molecular Biology, Medical Research Council, Cambridge CB2 0QH, UK; 2 Department of Microbiology, University of Washington, Seattle, WA 98195, USA

## Abstract

Chlorinated compounds are important environmental pollutants whose biodegradation may be limited by inefficient dechlorinating enzymes. *Dictyostelium* amoebae produce a chlorinated alkyl phenone called DIF which induces stalk cell differentiation during their multicellular development. Here we describe the identification of DIF dechlorinase. DIF dechlorinase is active when expressed in bacteria, and activity is lost from *Dictyostelium* cells when its gene, *drcA*, is knocked out. It has a K_m_ for DIF of 88 nM and K_cat_ of 6.7 s^−1^. DrcA is related to glutathione S-transferases, but with a key asparagine-to-cysteine substitution in the catalytic pocket. When this change is reversed, the enzyme reverts to a glutathione S-transferase, thus suggesting a catalytic mechanism. DrcA offers new possibilities for the rational design of bioremediation strategies.

## Highlights

► DIF-1 reductive dechlorinase is a GST-related protein ► A cysteine in the catalytic pocket is key for completing the reaction ► DIF dechlorination occurs through a glutathione-conjugated intermediate

## Introduction

Many different organisms can produce halogenated compounds, which are correspondingly widespread in nature. More than 4000 such compounds are now known, ranging from simple alkyl halides through to some of the most complicated of natural products. They are often thought to be produced for ecological purposes, as antifeeders or antibiotics, and, consistent with this idea, chlorinated compounds in particular are the source of important drugs including chloramphenicol, vancomycin, and spongistatin (Gribble, 1998, 2003, 2004).

These natural organohalides are not known to be particularly persistent in the environment or to produce ecological or human health problems, and in many cases enzymatic activities that catalyze their removal have been reported; it is thus interesting that several man-made organohalides are persistent and do cause problems. Well-documented cases of this include the chlorinated pollutants DDT, dioxins, and PCBs (Vasseur and Cossu-Leguille, 2006; Ritter et al., 1995). Certain of these compounds are banned or restricted in their use, and there can be significant costs in their disposal and in restoration of contaminated sites (http://www.who.int/ipcs/en; http://chm.pops.int/default.aspx).

The paradoxical persistence of man-made chlorinated compounds in the environment obviously relates to their intrinsic chemical stability, but also to the lack of efficient dechlorinating enzymes for them. Our current understanding of the dechlorination of man-made compounds comes mainly from the study of individual bacteria and bacterial collectives (Chaudhry and Chapalamadugu, 1991; Crawford and Ederer, 1999) that have been isolated from highly contaminated sites and selected for their ability to grow in the presence of the pollutant or used as a carbon or energy source (Parales and Haddock, 2004). This approach takes advantage of the promiscuous nature of some microbial enzymes, which can act on man-made compounds even though they may not be their natural substrates.

Dechlorination is often the limiting step in the degradation of chlorinated aromatics, because chlorine substitution of aromatic rings deactivates them for electrophilic attack and interferes with the action of dioxygenases, which are usually involved in ring cleavage. Under aerobic conditions, three different dechlorination mechanisms have been reported: hydrolytic, oxygen-dependent, and reductive dechlorination (Copley, 1997; Kurihara and Esaki, 2008; Fetzner and Lingens, 1994). The best-studied reductive dechlorinase is the tetrachlorohydroquinone dehalogenase PcpC, present in the pentachlorophenol degradation pathway of *Sphingomonas chlorophenolica* (Orser et al., 1993). PcpC catalyzes the conversion of tetrachlorohydroquinone (TCHQ) to trichlorohydroquinone and 2,6-dichlorohydroquinone using two molecules of glutathione per chlorine atom released (Xun et al., 1992; McCarthy et al., 1997). TCHQ dehalogenase has enzymatic and structural features that suggest an evolutionary relationship to the GST superfamily, as does LinD, a reductive dechlorinase involved in lindane degradation (Miyauchi et al., 1998; Vuilleumier and Pagni, 2002).

The kinetic properties of these dehalogenase enzymes are often very poor, however, with low turnover numbers, K_cat_, and K_m_ in the micromolar range in the best examples, perhaps reflecting the fact that the xenobiotic compounds upon which they are tested are not their natural substrates (Copley, 2009; Anandarajah et al., 2000; Fortin et al., 2006). Thus, dehalogenating enzymes with improved catalytic properties are desirable.

One possible source of more efficient dehalogenating enzymes is to find ones that have adapted to natural halogenated compounds over a long evolutionary history rather than those acting on more recently—in evolutionary terms—released man-made compounds.

A clear example is the mammalian tetraiodothyronine deiodinase, which reductively deiodinates thyroid hormone. This enzyme uses a highly reactive group, selenocysteine, present in the catalytic site to attack the carbon-iodine bond, and this is crucial in the deiodination reaction. The affinity of thyroid hormone deiodinases for their substrates is in the low nanomolar range (Bianco et al., 2002), and K_cat_ values are up to 0.4 s^−1^ (Salvatore et al., 1995).

**Figure 1:**
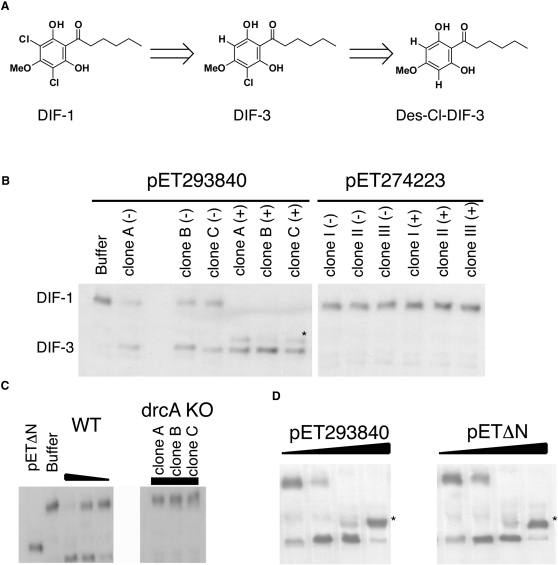
DIF-1 Reductive Dechlorination (A) DIF-1 and dechlorinated metabolites. (B) DIF-1 dechlorination assay using the cytosolic fraction of three clones of bacteria expressing DDB_G0293840 (clones A, B, and C) or DDB_G0274223 (clones I, II, and III) before (−) or after (+) induction with 1 mM IPTG. See also Table S1. (C) DIF-1 dechlorination assay of wild-type Ax2 (200–50 μg cytosolic fraction) and three independent *drcA* KO clones (200 μg cytosolic fraction). (D) DIF-1 dechlorination assay of purified protein (full-length DDB_G0293840) or ΔN allele. The asterisks mark the double-dechlorinated product.

The social amoeba *Dictyostelium discoideum* (Fets et al., 2010) produces more than a dozen chlorinated compounds during the multicellular stage of its life cycle (Kay et al., 1992). This stage is triggered by starvation and results in the production of a stalked fruiting body to aid dispersal of viable spores. The best known of these compounds is a chlorinated alkyl phenone called differentiation-inducing factor 1 (DIF-1) (Figure 1A), which induces stalk cell differentiation (Morris et al., 1987). The biosynthetic pathway for DIF-1 has been fully elucidated (Neumann et al., 2010; Austin et al., 2006; Kay, 1998; Thompson and Kay, 2000) and involves a dedicated pathway consisting of a “steely” polyketide synthase, an FADH-dependent halogenase, and a methyl transferase. The breakdown pathway is also partially known (Traynor and Kay, 1991; Morandini et al., 1995): the first step is a reductive dechlorination, for which a glutathione-requiring activity was identified in cell lysates more than 20 years ago (Nayler et al., 1992; Insall et al., 1992). However, the enzyme responsible has eluded full characterization and the gene encoding it has remained unknown to date.

Here we present the identification of the gene encoding DIF-1 dechlorinase (*drcA*) and experiments that led us to propose a catalytic mechanism for the enzyme. Although some deiodinases have been shown to also be active with chlorinated and brominated substrates (McTamney and Rokita, 2009), DIF dechlorinase is, to our knowledge, the first eukaryotic reductive dechlorinase characterized whose enzymatic mechanism has been elucidated with its natural substrate.

## Results

### Identification of the Gene Encoding DIF Dechlorinase

We combined in silico searches and biochemical purification to identify the gene encoding DIF dechlorinase. First, we ruled out a clear homolog of tetraiodothyronine deiodinase (Berry et al., 1991; Flatman, 2000), which is present in the *Dictyostelium* genome (*didA*; gene DDB_G0275741 at dictyBase; http://dictybase.org), because null mutants had normal DIF dechlorinase activity (not shown). Then we considered the possibility that DIF dechlorinase might be related to the glutathione S-transferase (GST) family, which contains enzymes capable of reductive dechlorination such as PcpC involved in the degradation of the xenobiotic pentachlorophenol by bacteria. No clear homolog of PcpC was detected among the 21 potential GSTs encoded in the *D. discoideum* genome (canonical domain composition GST_N+GST_C; see Table S1 available online), nor did any fall in the same class (beta class according to SuperFamily and GST_N/C according to NCBI) or cluster with it in phylogenetic analysis (Figure S1). We therefore turned to biochemical purification of the activity.

DIF-1 dechlorinase was partially purified from *Dictyostelium* lysates (see Experimental Procedures), and proteins present in the DIF dechlorinase-enriched fractions were identified by trypsin digestion and mass spectrometry. Of the more than 80 proteins thus identified, we focused on the two predicted GSTs (DDB_G0293840 and DDB_G0274223) as the biochemically most reasonable candidates. To test whether either of these encoded DIF-1 dechlorinase, they were cloned into bacterial expression vectors and the bacterial lysates were tested for DIF-1 dechlorination using an assay in which [^3^H]DIF-1 is converted to the monochlorinated DIF-3 and the products are extracted into an ethyl acetate phase and separated by TLC (Nayler et al., 1992).

As shown in Figure 1B, samples from bacterial cultures expressing DDB_G0293840 but not from cultures expressing DDB_G0274223 possess DIF-1 dechlorinating activity; this can be seen by the appearance of a slower-running TLC band at the same position as the monochlorinated molecule (DIF-3).

DDB_G0293840 is one of seven Ure2p-like GSTs present in the genome (Figure S1); we will refer to it as DIF dechlorinase or DrcA (*D*IF-1 *r*eductive de*c*hlorinase; gene *drcA*) interchangeably.

To test whether *drcA* encodes the DIF dechlorinating activity detected in *Dictyostelium* lysates, we knocked out the gene by homologous recombination. Lysates from the resulting null mutant strains (HM1467 and HM1468) lacked any detectable DIF dechlorinase activity at the slug stage (Figure 1C) or at any point during development (not shown), showing that *drcA* indeed encodes the DIF-1 dechlorinating activity detected previously and that it appears to be the only such enzyme encoded in the genome. Interestingly, the null mutant strain seems to develop normally and produced visually normal fruiting bodies.

A clear homolog of DrcA is encoded in the genome of the closely related social amoeba *Dictyostelium purpureum* (DPU_G0072892; http://www.dictybase.org). This gene was also expressed in bacteria, and after biochemical assay of the lysates was likewise found to encode a DIF dechlorinase (not shown).

### The N-Terminal Poly-Asn of DIF Dechlorinase Is Dispensable for Activity

DIF dechlorinase from *D. discoideum* is notable for having an N-terminal track of polyasparagine, a feature that it shares with the Ure2 protein from *Saccharomyces cerevisiae*, the founder member of the GST class to which it belongs. However, the *D. purpureum* protein lacks this feature, and as poly-Asn tracks are common in *D. discoideum* proteins and rarely have an established function, we tested whether this track is necessary for catalytic activity. A truncated version of DrcA, lacking the first 38 residues, ΔNdrcA, was expressed and found to be indistinguishable in activity from full-length DrcA (Figure 1D).

### Enzymatic Properties of DIF Dechlorinase

DIF dechlorination is strictly dependent on glutathione, and using intrinsic tryptophan fluorescence we estimated an affinity for glutathione at around 100 μM. Isothermal titration calorimetry (ITC) experiments confirmed an affinity constant of 102 ± 0.18 μM (Table 1).

Kinetic experiments at saturating glutathione and varying DIF-1 concentrations show that DrcA has a K_m_ for DIF-1 of around 88 nM, similar to the value originally described for the activity in *Dictyostelium* extracts (72 nM; Nayler et al., 1992). Assuming the DrcA protein purified from bacteria is fully active, DrcA has a turnover number of 6.7 s^−1^. These data represent a highly efficient dechlorinating enzyme with a K_cat_/K_m_ value 500 times higher than PcpC (Table 1).

When used at higher concentration or left for longer incubation times, DrcA can doubly dechlorinate DIF-1, again as originally described for the crude activity (Figure 1). The TLC band running slightly faster than monochlorinated DIF, corresponding to the doubly dechlorinated DIF, only appears after all DIF-1 has been converted into the monochlorinated form, suggesting a lower affinity of the enzyme for this chemical species.

### Cys54 Is Essential to Attack the GS-DIF Conjugate

The Ure2p-like class of GSTs, to which DIF dechlorinase belongs, is not very well characterized in terms of function, but structural data from several members are available and key residues involved in dimerization, substrate binding, and glutathione binding have been identified.

**Figure 2:**
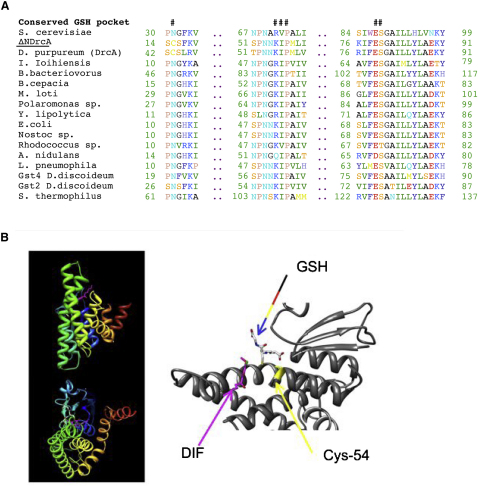
Structure of the Catalytic Pocket (A) Sequence alignment around the predicted glutathione binding site of DIF dechlorinase and other Ure2-like proteins from bacteria and fungi: *S. cerevisiae* (GenBank accession number 13399774), DrcA, *Idiomarina ioihiensis* (56459732), *Bdellovibrio bacteriovorus* (42523539), *Burkholderia cepacia* (46314031), *Mesorhizobium loti* (13474138), *Polaromonas* sp. (67907468), *Yarrowia lipolytica* (49651743), *Escherichia coli* (2495113), *Nostoc* sp. (17130470), *Rhodococcus* sp. (5911737), *Aspergillus nidulans* (40747210), *Legionella pneumophila* (54294869), Gst4, *D. discoideum* (28829738), Gst2, *D. discoideum* (20066256), *Streptococcus thermophilus* (55822716). # marks key conserved residues. See also Figure S1. (B) Three-dimensional model of DrcA, highlighting the predicted glutathione binding site and Cys54.

Figure 2A shows an alignment around the glutathione binding site of DIF-1 dechlorinase and several members of the Ure2p-like class (Morel et al., 2009; Marchler-Bauer et al., 2009), including the DrcA homolog from *D. purpureum*. DIF-1 dechlorinase, and its *D. purpureum* homolog, have five out of six key residues conserved, but in the position where all other members have an asparagine residue, they have a cysteine. Although cysteine residues occur around this position in other classes of GSTs, such as the zeta class, DIF dechlorinase is the only Ure2p-like member identified so far with this substitution. A 3D model based on the closest crystal structure available shows that this cysteine is located close to the glutathione-binding pocket (Figure 2B). The same structural features are predicted in the *D. purpureum* homolog.

**Figure 3:**
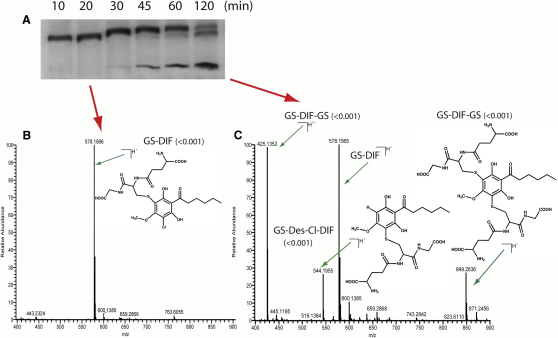
Identification of DIF-1 Metabolites (A) Analytical time course of the aqueous phase-partitioning metabolites produced by purified ΔNC16NDrcA mutant protein. (B and C) Mass spectrum survey scans of samples corresponding to 20 min (B) or 120 min (C) after incubation with purified ΔNC16NDrcA. Main ions were fragmented and further analyzed (Figure S2); the deduced chemical structures are shown. The inaccuracy of m/z for each compound is shown in parentheses.

To test whether this “natural substitution” of an asparagine by a cysteine is important for enzymatic activity, we made a point mutant replacing this cysteine residue of DrcA with an asparagine (Cys54 in wild-type DrcA, Cys16 in the ΔNdrcA allele). This mutated version (ΔNC16NDrcA) was expressed and purified to homogeneity and tested in the standard DIF-1 dechlorination assay. We found that although DIF-1 was consumed in the assay, DIF-3 was not produced and no products appeared in the organic phase when the reaction mix was extracted with organic solvents. Instead, a radioactive product appeared in the aqueous phase, indicating that a more polar compound was formed. To visualize this compound, we separated the radioactive compounds from the aqueous phase by TLC with more polar solvents and, as shown in Figure 3A, found that a single product appears at short incubation times and, later on, a second, slower-running molecule appears and accumulates concomitantly with the disappearance of the first one. The accumulation of this second species is accompanied by the appearance of a third less intense band.

To identify the products made by the mutant enzyme, the aqueous-phase products were desalted and introduced directly into an LTQ-Orbitrap XL mass spectrometer using an offline static electrospray needle. MS survey scans were measured from 155 to 1000 m/z. The most prominent ions were selected, fragmented, and analyzed in the Orbitrap (Figure 3).

A novel main peak of m/z 578.1566 (MH^+^) was detected in the earliest sample taken, which is enriched for the first chemical species produced. This was monochlorinated, as indicated by the characteristic chlorine isotope cluster with the main and +2 peaks in a ratio of 3:1 (Figure S2). Further fragmentation allowed this compound to be identified as glutathione-conjugated DIF (GS-DIF) (Figure S2). The second intense band was not chlorinated, and was identified as a double-glutathione conjugate of DIF appearing as peaks of m/z 849.2636 (MH^+^) and m/z 425.1352 (MH^2+^) (GS-DIF-GS; Figure S2); the third chemical species appears to be associated with a peak of m/z 544.1955 (MH^+^) and is also not chlorinated. This peak is not observed at early time points but only after GS-DIF-GS has started to accumulate, and is clearly present at late time points (Figure 3; Figure S2). The compound was identified as a breakdown product of double glutathione-conjugated DIF, likely arising from spontaneous cleavage of one thioether bond, where the sulfur atom remains attached to the glutathione backbone.

These findings strongly suggest that the DIF-1 dechlorination reaction goes through a glutathione-conjugated DIF intermediate that requires Cys54 for resolution. This model is supported by the observation that GS-DIF produced by point mutant ΔNC16NDrcA is readily converted to monochlorinated DIF (DIF-3) when treated with wild-type ΔNDrcA (Figure S3).

## Discussion

DIF-1 is unusual among natural chlorinated compounds in being a developmental signal in the organism that produces it, rather than acting as an ecological effector. As DIF is active at low nanomolar concentrations and levels need to be carefully regulated during development, it is perhaps not surprising that *Dictyostelium* cells have evolved an efficient means of inactivation. The enzyme carrying out this inactivation, DIF dechlorinase, was first detected in cell lysates 20 years ago, and has now been identified.

**Figure 4:**
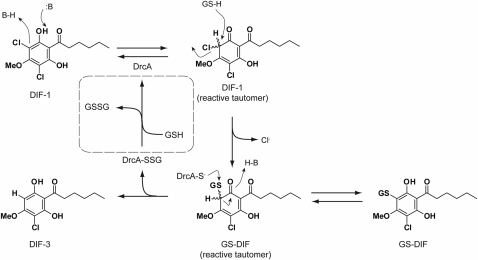
Proposed Mechanism of Action for DrcA See also Figure S3.

DIF dechlorinase is related by sequence to the superfamily of glutathione S-transferases, as is PcpC, the best-studied bacterial reductive dechlorinase. Although sequence similarity between PcpC and DIF dechlorinase is very low, we propose that they share a similar mechanism of action. In the first stage of this mechanism (Figure 4), DrcA acts in an analogous manner to prototypical GSTs: a glutathione molecule attacks the carbon-chlorine bond to create a GS-DIF conjugate. This conjugate is then attacked by a cysteine residue of the protein, releasing the monodechlorinated product (DIF-3) and leaving the cysteine conjugated to glutathione. To complete the cycle, the enzyme reacts with another glutathione molecule that renders oxidized glutathione and regenerates the enzyme.

This mechanism resembles the action of GSTs as detoxifying enzymes, where the protein activates the glutathione and puts the substrate in the proper orientation to allow the GS^∗^ to perform a nucleophilic attack leading to conjugate formation. In the present case, the enzyme likely promotes formation of a nonaromatic tautomer of DIF-1, thereby activating the chlorine for displacement by GSH in an S_N_2-like fashion. This mechanism is analogous to those proposed for TCHQ dehalogenase (Warner and Copley, 2007) and BhpK (Tocheva et al., 2006). In the second stage of the reaction, the breakdown of the GS-DIF conjugate is achieved by a reactive cysteine located close to the catalytic site (Figure 2B). Again, the electronics of the reaction suggest the intermediacy of a nonaromatic tautomer of GS-DIF. We have been able to identify the catalytic cysteine residue and, using a point mutant in this residue, can “freeze” the reaction after the first step of conjugate formation to observe the GS-DIF intermediate. It is the presence of this cysteine that allows DrcA to go beyond being just a glutathione-conjugating enzyme and become a reductive dechlorinase.

Interestingly, ΔNC16NDrcA must be able to accommodate the conjugate GS-DIF to attack the second carbon-chlorine bond, because we are able to detect a GS-DIF-GS product by mass spectrometry. This observation suggests DrcA contains a large substrate pocket.

Because DIF dechlorinase appears to be catalytically much more efficient than any reductive dechlorinase so far described (K_m_ of around 88 nM and a turnover number of at least 6.7 s^−1^) and may have a relatively large substrate pocket that could accommodate a range of chemicals, it is a promising starting point for the development of efficient enzymes to dechlorinate xenobiotics.

## Significance

**DIF dechlorinase, which inactivates a natural signal molecule used by the social amoeba *Dictyostelium* in its development, was first detected in cell lysates more than 20 years ago and has now been identified. The enzyme performs a reductive dechlorination of the aromatic ring of DIF using glutathione and is closely related to glutathione S-transferases. The catalytic mechanism can be abrogated by a single amino acid change so that the enzyme produces a DIF-glutathione conjugate instead of dechlorinated DIF, thus suggesting a catalytic mechanism.**

**Aromatic halogenated compounds are widely used in industry, agriculture, and medicine and some persist in the environment, causing serious problems. Known dechlorinating enzymes for pollutants such as DDT, PCBs, and (2,4-dichlorophenoxy)acetic acid are inefficient, presumably because these compounds have only recently appeared in the environment. In contrast, DIF dechlorinase, acting on its natural substrate, is much more efficient and may therefore provide lessons from which more efficient dechlorinating enzymes can be designed. In addition, the identification of the DIF dechlorinase gene will allow the role of DIF dechlorination to be explored in *Dictyostelium* development.**

## Experimental Procedures

### *Dictyostelium* Growth and Molecular Genetics

Ax2 (Kay Laboratory strain, DBS0235521; http://www.dictybase.org) was used as wild-type axenic strain. *Dictyostelium* cells were grown shaken in axenic medium (Foremedium) to a density of 4–6 × 10^6^ cells/ml (mid-log phase) at 22°C and washed twice in KK2 buffer (20 mM potassium phosphate [pH 6.2]) supplemented with 2 mM MgSO_4_ prior to any manipulation. For normal development, cells were plated on KK2 agar (1.8%, Oxoid L28) at a density of 8 × 10^5^ cells/cm^2^.

To induce DrcA activity, 2 l of *D. discoideum* cells at 2.5 × 10^7^ cell/ml in KK2 buffer was pulsed every 6 min for 10 hr with cAMP (80 nM final concentration). After pulsing, a single shot of 100 nM DIF-1 was added and the cells were incubated for 45 min and then harvested, and the pellets were frozen in liquid nitrogen and stored at −80°C until use. Alternatively, developmental plates were prepared and multicellular structures were harvested at different time points (14 hr for slugs), and pellets were frozen in liquid nitrogen and stored at −80°C until use.

A null mutant of *drcA* was constructed by homologous recombination resulting in deletion of the ORF and its replacement by a blasticidin resistance cassette. KO plasmid pKO229820 was constructed with upstream and downstream homology regions amplified by PCR from genomic DNA using the pair of primers (Fw229820KOUP 5′-CCAGGTACCTTTAAACCTGGTCATTTTG-3′/Rev220920KOUP 5′-GCAACTCCTAAGCTTCTTCCATCATCAGC-3′) and (Fw229820KODWN 5′-CGTTGGGATCCTACAATGTCTTCAAGACC-3′/Rev229820KODWN 5′-CAACAGAAACAATACCGGCGGCCGCAAATTCCC-3′), respectively, and then cloned into the appropriate restriction sites of pLPBLP (Faix et al., 2004). Transformants were selected in axenic media with blasticidin (10 μg/ml) and KO were mutants identified by PCR using Fw229820/RevKOcheck (5′-CTTGTTCAAAAATGATTCTGTAGTTGGCC-3′) and FwKOcheck (5′-CCACACACGTAAATAAGAATGCCAAGG-3′/Rev229820). Three independent clones were kept.

### DIF Dechlorinase Assay

DIF dechlorinase was assayed as described, using custom-synthesized [^3^H]methoxy-DIF-1 as substrate and TLC separation of products (Nayler et al., 1992). Samples were applied to Whatman LK6D silica gel TLC plates, which were developed with hexane:ethyl acetate:acetic acid (60:40:2 by volume). The chromatograms were sprayed with Enhance (PerkinElmer), air dried, and exposed at −70°C to Amersham Hyperfilm MP (GE Healthcare). Substrate conversion was quantified by scraping the appropriate bands from the TLC plates into 0.5 ml ethanol and 4 ml scintillation liquid, followed by scintillation counting; 1 U DIF-1 converting activity is defined as 1 pmol/min. The amount of protein or the incubation time was adjusted to ensure that no more than 30% was converted. The concentration of nonradioactive DIF-1 was calculated by UV absorbance using its extinction coefficient (^DIF-1^E _277nm_ = 10,600) reported by Masento et al. (1988). Kinetic parameters were obtained using purified ΔNDrcA diluted in reaction buffer supplemented with 1 mg/ml BSA to 18 ng/ml (628 pM) in the assay with substrate concentrations of 50–800 nM and initial velocities (less than 30% substrate metabolized) calculated. Prism 5 (GraphPad Software) was used to fit a Michaelis-Menten model and calculate kinetic parameters.

### Protein Purification

Frozen pellets were resuspended in buffer A (20 mM HEPES [pH 7.4], 50 mM KCl, 2 mM EDTA, 2 mM dithiothreitol, 10% glycerol, plus complete EDTA-free protease inhibitor cocktail from Roche) and the cells were pressure homogenized using an Emulsiflex-C5 (Avestin Europe GmbH) according to the manufacturer's recommendations. The high-speed supernatant was prepared by centrifugation at 100,000 × g for 1 hr. The soluble fraction remaining after ammonium sulfate precipitation (1.5 M final concentration) of the high-speed supernatant was loaded onto an S-butyl Sepharose Fast Flow column (GE Healthcare) and eluted with a linear gradient of salt in buffer A using an AKTA FPLC system (GE Healthcare). Active fractions were loaded onto a DE-52 (Whatman) column and eluted with a linear gradient of KCl in buffer A. Active fractions were concentrated and loaded onto a gel filtration column (HiLoad 26/60 Superdex 200) in buffer A. Active fractions were concentrated and resolved by SDS gel electrophoresis, and fractions from 25 kDa to 50 kDa were used for protein identification by mass spectrometry following tryptic digestion.

### Bacterial Expression

Coding sequences of genes of interest were PCR amplified and cloned into pET28-c NcoI/HindIII sites to generate a C-terminal 6×His-tagged protein. Pairs of primers used were Fw229820 (5′-AACCATGGATTGTATTAATAACTATAATAATAATAATAATAATAATAATAATAATAATAAAATTATGATACC-3′)/Rev229820 (5′-TTAAAGCTTTAATTCTTGATTATCTAATCTTTTTAATTTTCTAACTC-3′), Fw274223 (5′-AACCATGGTAGAAATTAATAATAAAGTAGATTATATATTC-3′)/Rev274223 (5′-ATCAAGCTTTAAAACAACATTTTCAATACC-3′), and FwΔN (5′-GGAACCATGGAAGGAGTTGCAGACTATCAAGTTTATGG-3′)/Rev229820 to amplify full-length DDB_G0293840, DDB_G0274223, and the truncated N-terminal allele of DDB_G0293840, respectively; the corresponding plasmids pET293840, pET274223, and pETΔNdrcA were transformed into the BL21 (DE) STAR bacterial strain (Invitrogen). Bacterial cultures were induced with 1 mM isopropyl-β-d-thio-galactoside (IPTG) for 3 hr before harvesting, and cell extracts and soluble fractions were prepared as described for *Dictyostelium* cells. The expressed protein was purified using Ni-NTA resin (QIAGEN) following manufacturer recommendations. The eluate was concentrated and the protein was further purified by gel filtration (HiLoad 26/60 Superdex 200). The point mutation C16N was introduced by PCR using the primers FwC16N (5′-TACCATGGAAGGAGTTGCAGACTATCAAGTTTATGGATTTTATACATCTAATTCATTT-3′)/Rev229820, using pETΔNdrcA as template.

### ITC and Tryptophan Intrinsic Fluorescence

ITC and tryptophan intrinsic fluorescence (TIF) were performed with purified protein. TIF was performed using 100 μM protein and concentrations of reduced glutathione spanning 0–2 mM. ITC was performed using VP-ITC (MicroCal) with 5 mM glutathione in the syringe and 150 μM protein.

### Identification of Glutathione-Conjugated DIF-1 Metabolites

The DIF dechlorinase assay was carried out using ΔNC16NDrcA protein and a modified reaction buffer without glycerol. The reaction was stopped at different time points with a mixture of ethyl acetate:hexane:acetic acid (Nayler et al., 1992).

To visualize the products, radioactive DIF-1 was used as substrate and aqueous-phase compounds were separated by TLC with butanol:acetic acid:water 12:3:5. For identification of products, nonradioactive DIF-1 was used as substrate. Then the aqueous phases were desalted using C18 Zip-Tip (Millipore) and the eluate was directly introduced into an LTQ-Orbitrap XL (Thermo Fisher Scientific) by an offline static electrospray needle from MasCom. MS survey scans were measured from 155 to 1000 m/z, and selected ions were fragmented and analyzed in the Orbitrap.

### Sequence Analysis

Proteins coded by the *D. discoideum* genome carrying a GST domain were obtained from the SUPERFAMILY database (http://supfam.cs.bris.ac.uk/superfamily/index.html) and Pfam database (Pfam version 24.0): GST_N (PF02798) and GST_C (PF00043) (http://pfam.sanger.ac.uk). Alignments were made using default parameters using CLUSTALW software (http://www.ebi.ac.uk/tools/msa/clustalw2), and Blastp searches were made using default parameters at NCBI (http://blast.ncbi.nlm.nih.gov/blast.cgi) and dictyBase (http://dictybase.org/tools/blast). Sequences of representative members of different cytosolic GST were obtained from the UniProt database (http://www.uniprot.org) as follows.

Beta class: P44521, Q9PE18, P45875, P81065, P15214, Q2QCP3, P0A9D2, ZP_00054555, O33705. Phi class: P42769, P46422, Q9ZP62, P12653, P30111. Lambda class: Q9LZ07, P49248, Q9M2W2. Omega class: P78417, Q9Z339, P34345. Tau class: O04941, O24595, Q9ZP61, Q10CE7. Alpha class: P10648, P08263, P00502, P13745, P24472, P08515, P30114, B5AK46. Sigma class: O60760, P46429, O35543, P41043, P46088, Q9NJQ6, O60760, O35543. Mu class: P09488, P46419, P04905, P20136. Pi class: P46427, P09211, P80031, P46425. Zeta class: O43708, O04437, Q9ZVQ3. Ure2 class: P23202, Q9RBP3. Theta class: Q2NL00, P0CG29, P21161, P43387. Delta/epsilon class: Q93113, Q9VG96, Q94999, Q7KIF1, Q8WQJ9, Q7KK90, Q9GNE9. Glutaredoxin2 class: P0AC59. GST-like domain of elongation factor 1-γ: P29547. Chloride intracellular channel 1: O00299. Microsomal prostaglandin E synthase-2: Q9N0A4. Other GSTs: Q51948, P30347, Q03520, Q8UJG9, Q8DMB4, Q5N5D3, P27457, Q937X0, P30568, Q50LH1, Q6VNV2, Q6WMS8, Q5TGI0, Q8MU52, Q7D2W7.

### Three-Dimensional Model of DIF Dechlorinase

Three-dimensional modeling of DrcA was performed by the I-TASSER server (http://zhanglab.ccmb.med.umich.edu/i-tasser; Roy et al., 2010) using full-length and N-terminal truncated allele sequences from *D. discoideum* and the DrcA sequence from *D. purpureum*. The output model from the server was subsequently refined with simulated annealing/molecular dynamics and conjugate gradient minimization with no experimental energy terms using CNS (Brünger et al., 1998). The refined model was used for manual docking of the DIF molecule in putative binding sites. Another cycle of refinement was used in order to prevent steric clashes between the protein and ligands. Superposition as well as 3D analysis was performed with Chimera (Pettersen et al., 2004).

## Figures and Tables

**Table 1:** Kinetic Parameters of DrcA and Other Reductive Dechlorinases

Kinetic Parameters	K_a_ GSH (μM)	K_m_ Substratum (nM)	K_cat_ Substratum (s^−1^)	K_cat_/K_m_ (M^−1^ s^−1^)
DrcA (DIF-1)	102	88 ± 17.5	6.7	76,000,000
PcpC (TriCHQ)	32	20,000	3	150,000
BphK (3-Cl HOPDA)	110	17,000	0.16	10

Values for PcpC and BphK are taken from Anandarajah et al. (2000) and Fortin et al. (2006).

## Supplemental Information

Document S1. One Table and Three Figures
