# Supplementary material for: Identification of a Eukaryotic Reductive Dechlorinase and Characterization of Its Mechanism of Action on Its Natural Substrate
Source: Chem Biol. 2011 Oct 28;18(10):1252–60. doi: 10.1016/j.chembiol.2011.08.003 (PMC3205185; doi:10.1016/j.chembiol.2011.08.003)
Supplement: Document S1. One Table and Three Figures [file mmc1.pdf]

Chemistry & Biology, *18*

Supplemental Information

Identification of a Eukaryotic Reductive Dechlorinase and Characterization of Its Mechanism of Action on Its Natural Substrate

Francisco Velazquez, Sew Yu Peak-Chew, Israel S. Fernández, Christopher S. Neumann, and Robert R. Kay

<u>Inventory of Supplemental Information</u>

Table S1. Comprehensive list of predicted ORFs in *D. discoideum* genome with a prototypic cytosolic GST domain architecture (GST_N+GST_C) according to Pfam or Superfamily databases. Numbers correspond to e-value assigned by each database to each domain (shaded cells correspond to values above the threshold). NCBI* correspond to significant assignation of GST domains to specific class at NBCI conserved domains database. Putative gst6 and gst21 are duplicated in the genome of Ax4 wild type strain. DrcA/Gst3 is underlined to highlight it. Related to Figure 1.

Figure S1. A) Phylogenetic tree of all putative cytosolic GSTs from *D. discoideum* and representative members of all SCOP GST classes (SCOP 52862/SCOP 47617). B) Phylogenetic tree of all putative cytosolic GSTs from *D. discoideum* (SCOP 52862/ SCOP 47617). Numbering of *D. discoideum* GSTs given in Table S1. * marks DrcA. Related to Figure 2.

Figure S2. Fragmentation products of ion 578.1568 (A), 425.1352 (B) and assigned structures for the main peaks. The characteristic chlorine isotope cluster is not observed due the tight selection of only one isotopic form. C) Mass spectrum survey scan of sample corresponding to 45 minutes of incubation with ΔNC16NDrcA. D) Zoom of Mass spectrum survey of peak 578.1568 (TOP) showing characteristic chlorine isotope cluster with main and +2 peaks in an approximate 3:1 ratio. These are absent in the non-chlorinated compound in peak 849.2636 (Bottom). Related to Figure 3.

Figure S3. GS-DIF is a substrate for the wild type enzyme. Lane A, aqueous phase TLC showing the GS-DIF conjugated produced by ΔNC16NDrcA; a fraction of this phase was later incubated either with ΔNC16NDrcA (lane 1) or WT ΔNDrcA (lane 2). Related to Figure 4.

Table S1. Putative cytosolic GSTs coded in *D. discoideum* genome.

	ID	Pfam GST_N	Pfam GST_C	Superfam Thio-Like	Superfam GST_C	NCBI class N-terminus*	NCBI class C-terminus*
Gst1	DDB_G0278155	2.8e-14	3.2e-06	2.21e-25	2.92e-33	ZETA	ZETA
Gst2	DDB_G0276351	5.1e-09	3.1e-10	4.75e-21	1.12e-33	URE2p	URE2p
Gst3 DrcA	DDB_G0293840	2.4e-06	3.8e-09	7.78e-21	6e-33	URE2p	URE2p
Gst4	DDB_G0271892	1.4e-09	3.9e-12	8.04e-20	1.67e-29	URE2p	URE2p
Gst5	DDB_G0287793	0.00011	5.7e-14	6.9e-18	3.55e-26	THETA	THETA
Gst6	DDB_G0272632/ DDB_G0274081	1.3e-10	1.4e-09	1.01e-17	1.67e-21	SIGMA	SIGMA
Gst7	DDB_G0274223	6.2e-07	1.1e-09	7.33e-17	1.5e-28	Thioredoxin -like spFAM	URE2p
Gst8	DDB_G0280881	2e-08	8.4e-08	7.78e-17	4.37e-28	URE2p	URE2p
Gst9	DDB_G0274705	4.6e-07	4.9e-09	1.92e-16	4.37e-26	URE2p	URE2p
Gst10	DDB_G0293122	0.0002	8.1e-09	6.28e-16	2.09e-22	THETA	THETA
Gst11	DDB_G0283575	3.3e-10	1.3e-10	1.56e-15	8.63e-25	SIGMA	SIGMA
Gst12	DDB_G0286341	--	3.2e-10	1.12e-14	3.48e-35	--	ECM4
Gst13	DDB_G0292030		0.027	2.11e-13	1.29e-21	--	ECM4
Gst14	DDB_G0268138	7.4e-06	1.8e-08	5.85e-13	5.43e-20	SIGMA	SIGMA
Gst15	DDB_G0280317	3e-06	4.9e-06	4.83e-12	1.77e-20	Thioredoxin -like spFAM	SIGMA
Gst16	DDB_G0278997	--	1.4e-05	3.45e-11	2.37e-20	--	ECM4
Gst17	DDB_G0278999	--	0.00023	6.12e-11	1.81e-19	--	gst_C spFAM
Gst18	DDB_G0271958	0.0014	1.7e-09	1.73e-10	1.17e-16	Thioredoxin -like spFAM	gst_C spFAM
Gst19	DDB_G0274263	0.00093	4.9e-10	3.67e-10	1.53e-22	SIGMA	SIGMA
Gst20	DDB_G0276899	--	0.0031	1.48e-08	3.96e-11	METAXIN	METAXIN
Gst21	DDB_G0273153/ DDB_G0273923	0.35	5.7e-07	0.00206	6.96e-16	--	gst_C spFAM
--	PcpC	1.5e-11	2.1e-13	1.32e-12	7.24e-20	GST_N	GST_C

Comprehensive list of predicted ORFs in *D. discoideum* genome with a prototypic cytosolic GST domain architecture (GST_N+GST_C) according to Pfam or Superfamily databases. Numbers correspond to e-value assigned by each database to each domain (shaded cells correspond to values above the

threshold). NCBI* correspond to significant assignation of GST domains to specific class at NBCI conserved domains database. Putative gst6 and gst21 are duplicated in the genome of Ax4 wild type strain. DrcA/Gst3 is underlined to highlight it. Related to Figure 1.

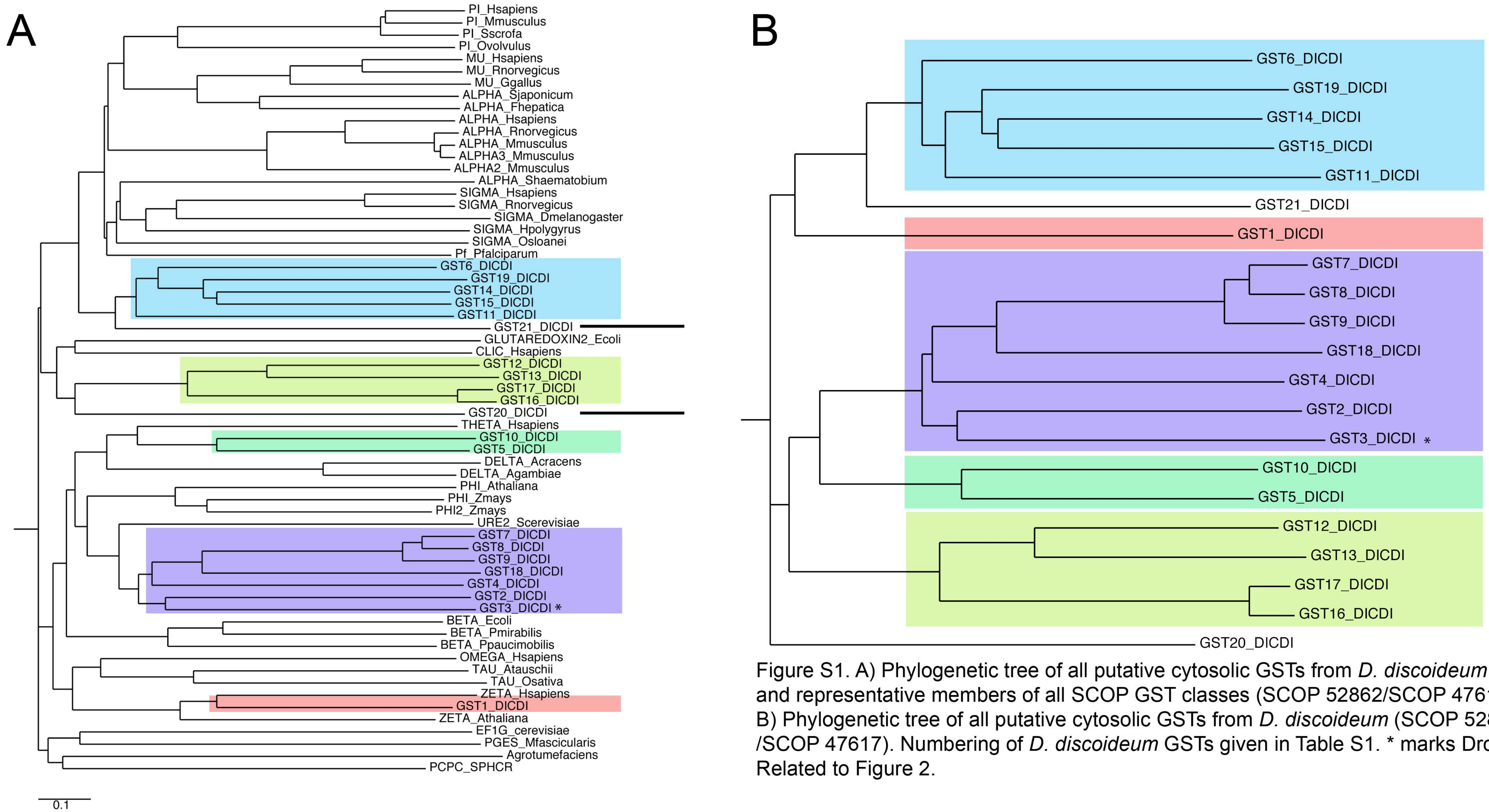

Figure S1. A) Phylogenetic tree of all putative cytosolic GSTs from *D. discoideum* and representative members of all SCOP GST classes (SCOP 52862/SCOP 47617). B) Phylogenetic tree of all putative cytosolic GSTs from *D. discoideum* (SCOP 52862 /SCOP 47617). Numbering of *D. discoideum* GSTs given in Table S1. * marks DrcA. Related to Figure 2.

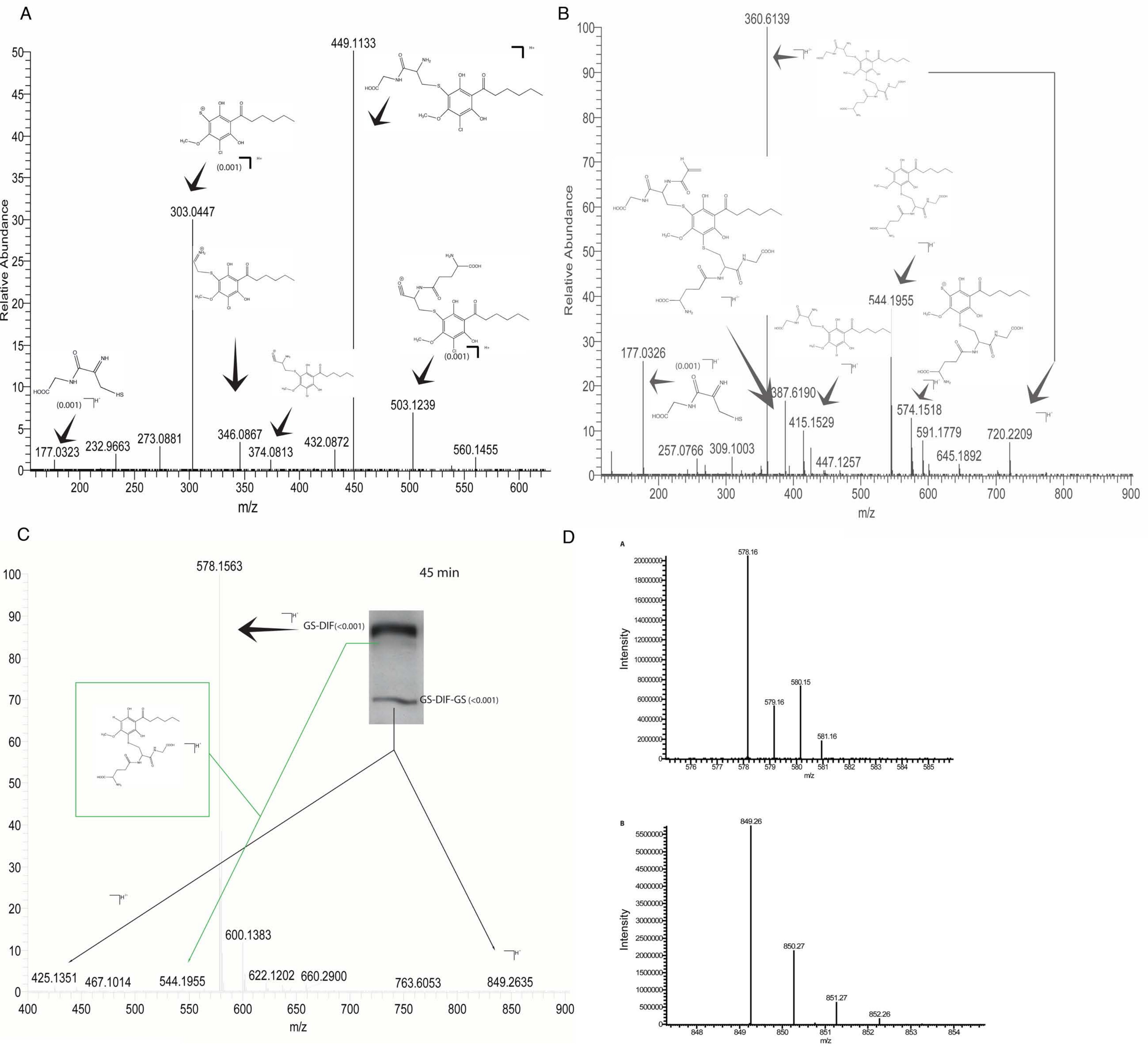

Figure S2. Fragmentation products of ion 578.1568 (A), 425.1352 (B) and assigned structures for the main peaks. The characteristic chlorine isotope cluster is not observed due the tight selection of only one isotopic form. C) Mass spectrum survey scan of sample corresponding to 45 minutes of incubation with ΔNC16NDrcA. D) Zoom of Mass spectrum survey of peak 578.1568 (TOP) showing characteristic chlorine isotope cluster with main and +2 peaks in an approximate 3:1 ratio. These are absent in the non-chlorinated compound in peak 849.2636 (Bottom). Related to Figure 3.

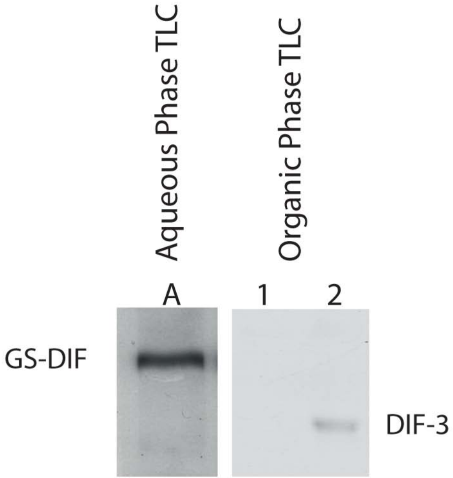

Figure S3. GS-DIF is a substrate for the wild type enzyme. Lane A, aqueous phase TLC showing the GS-DIF conjugated produced by ΔNC16NDrcA; a fraction of this phase was later incubated either with ΔNC16NDrcA (lane 1) or WT ΔNDrcA (lane 2). Related to Figure 4.
